# Generation and Export of Red Blood Cell ATP in Health and Disease

**DOI:** 10.3389/fphys.2021.754638

**Published:** 2021-11-05

**Authors:** Timothy J. McMahon, Cole C. Darrow, Brooke A. Hoehn, Hongmei Zhu

**Affiliations:** Department of Medicine, Division of Pulmonary, Allergy, and Critical Care Medicine, Durham VA and Duke University Medical Centers, Durham, NC, United States

**Keywords:** transfusion, blood flow, sepsis, hypoxia, endothelial cells

## Abstract

Metabolic homeostasis in animals depends critically on evolved mechanisms by which red blood cell (RBC) hemoglobin (Hb) senses oxygen (O_2_) need and responds accordingly. The entwined regulation of ATP production and antioxidant systems within the RBC also exploits Hb-based O_2_-sensitivity to respond to various physiologic and pathophysiologic stresses. O_2_ offloading, for example, promotes glycolysis in order to generate both 2,3-DPG (a negative allosteric effector of Hb O_2_ binding) and ATP. Alternatively, generation of the nicotinamide adenine dinucleotide phosphate (NADPH) critical for reducing systems is favored under the oxidizing conditions of O_2_ abundance. Dynamic control of ATP not only ensures the functional activity of ion pumps and cellular flexibility, but also contributes to the availability of vasoregulatory ATP that can be exported when necessary, for example in hypoxia or upon RBC deformation in microvessels. RBC ATP export in response to hypoxia or deformation dilates blood vessels in order to promote efficient O_2_ delivery. The ability of RBCs to adapt to the metabolic environment *via* differential control of these metabolites is impaired in the face of enzymopathies [pyruvate kinase deficiency; glucose-6-phosphate dehydrogenase (G6PD) deficiency], blood banking, diabetes mellitus, COVID-19 or sepsis, and sickle cell disease. The emerging availability of therapies capable of augmenting RBC ATP, including newly established uses of allosteric effectors and metabolite-specific additive solutions for RBC transfusates, raises the prospect of clinical interventions to optimize or correct RBC function *via* these metabolite delivery mechanisms.

## Red Blood Cells and Metabolic Homeostasis

Red blood cells (RBCs) and their exquisitely evolved, oxygen-carrying hemoglobins (Hbs) allow multicellular organisms to respire *via* the finely tuned mass delivery of oxygen (O_2_) to the tissues and the elimination of CO_2_ shuttled from tissues to the lungs. Hb and the RBC serve metabolic responsiveness not only in their function as mobile reservoirs for O_2_ and CO_2_, but also *via* the regulated release of vasoactive mediators. Such mediators include the central metabolite ATP and *S*-nitrosothiols (SNOs), which are derivatives of nitric oxide (NO, a “third gas” in the respiratory cycle) (Sonveaux et al., 2007). Here we review the generation and function of RBC-derived ATP in health and disease. This pathway has been increasingly informed by recent ‘omics and other new findings, and made increasingly tractable by recently developed or repurposed therapeutics.

## ATP Generation by Red Blood Cells in Context

**FIGURE 1:**
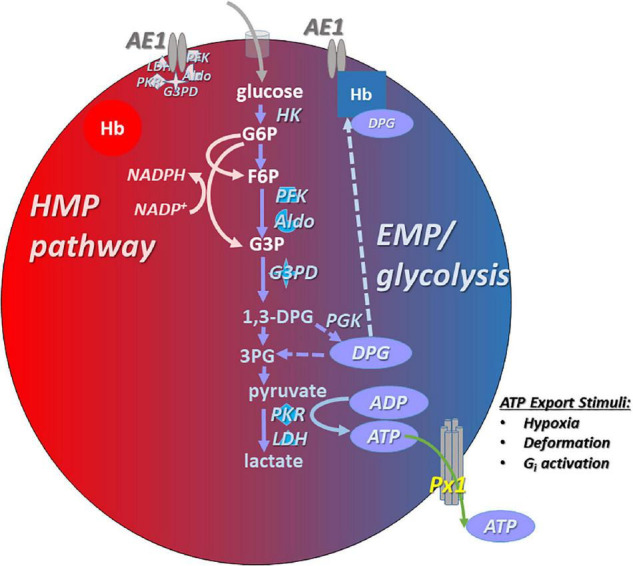
Simplified schema describing ATP generation and export from human RBCs. Glycolysis *via* the Embden–Meyerhof pathway (*EMP*; in *blue* on right side of the RBC cartoon) is the principal path to ATP generation in RBCs. EMP activity and ATP (and DPG) generation are favored under low-oxygen conditions, in which membrane-resident AE1 (anion exchanger 1, aka band 3) binds deoxygenated hemoglobin (Hb), freeing up the glycolytic enzyme complex (metabolon) that associates with AE1 when not bound by Hb (i.e., when Hb is oxygenated; pink symbols in upper left of the cartoon RBC). *ATP* is exported basally, and hypoxia or RBC deformation increase *ATP* export. Pannexin 1 (*Px1*) appears to be necessary for O_2_- and deformation-responsive export of *ATP* from the RBC. The exported *ATP* can act to limit the adhesivity of RBCs to endothelial cells, effect vasodilation, and may have anti-permeability (Kolosova et al., 2008) and other roles. In oxygenated RBCs, activity of the hexose monophosphate (*HMP*) pathway is favored because *EMP enzymes* are sequestered to AE1, freeing up shared substrate and generating *NADPH* that provides the reducing equivalents necessary to power antioxidant systems in the face of high O_2_ flux.

Red blood cells produce energy by glycolysis only (Figure 1), *via* two competing branches (Rogers et al., 2009): the Embden–Meyerhof pathway (EMP) which generates ATP, and the hexose monophosphate pathway (HMP), the sole route for recycling nicotinamide adenine dinucleotide phosphate (NADPH), which powers the thiol-based antioxidant system critical for homeostasis in the O_2_-rich RBC (Siems et al., 2000). EMP vs. HMP dominance is gated or toggled as a function of the assembly of an EMP protein complex (or “metabolon”) upon the cytoplasmic domain of the band 3 membrane protein [cdB3, also known as anion exchanger 1 (AE1)] (Low et al., 1993; Messana et al., 1996; Sterling et al., 2001; Bruce et al., 2003; Barvitenko et al., 2005; Campanella et al., 2005; Chu and Low, 2006; Chu et al., 2008). Metabolite flux through EMP vs. HMP oscillates depending on the Hb conformation (oxygenation state) and cdB3 phosphorylation. RBC deoxygenation promotes the generation of ATP (Figure 1), while full oxygenation of RBCs promotes NADPH generation (Rogers et al., 2009, 2013; Kirby et al., 2014). Rogers and coworkers showed that RBC antioxidant systems fail when HMP flux is blunted by altered cdB3 protein assembly/phosphorylation caused by aberrant Hbs or hypoxia (Rogers et al., 2009, 2013; Ferru et al., 2011). In experiments using dehydroepiandrosterone (DHEA) to mimic glucose-6-phosphate dehydrogenase (G6PD) deficiency in RBCs, both NADPH generation (as expected) and ATP export were depressed, underlining the importance of redox maintenance in responsive ATP export capacity (Subasinghe and Spence, 2008).

## ATP Acts Both Within and Beyond the Red Blood Cell to Modulate Blood (Red Blood Cell) Flow

The determinants of RBC deformability are numerous and complex. ATP generated within the RBC is pivotal because of its importance in the energy-dependent maintenance of ionic and structural homeostasis within RBCs as they experience fluctuating mechanical and chemical stresses during circulation (McMahon, 2019). In addition, the ATP exported from RBCs appears to act in several modes that subserve efficient blood flow, including vasodilation in proportion to the degree of hypoxia, inhibition of intercellular adhesion, and the prevention of unwanted capillary permeability (Zhu et al., 2011; Kirby et al., 2021). Measurement of plasma ATP is challenging. Changes in plasma ATP under conditions known to promote ATP export from RBCs and to promote blood flow are on the order of ∼60 nM [e.g., from ∼95 nM basally to 155 nM in hypoxia in mice, or from ∼62 nM basally to 104 nM in hypoxia in young (∼23-years old), healthy human subjects (Kirby et al., 2012)]. The anti-adhesive effects of ATP exported from human RBCs appear to involve specific receptor/ligand/counter-receptor systems. We found that antibodies to either the RBC LW/ICAM-4 adhesion receptor on RBCs, or antibodies to the cognate α_v_β_3_ integrin receptors on endothelial cells (ECs) prevented the proadhesive effect of inhibiting ATP export (Zhu et al., 2011). These findings indicate that ICAM-4 on RBCs and α_*v*_β_3_ integrin are responsible for the ATP-sensitive modulation of intercellular adhesion. Antagonizing ICAM-4 on RBCs deficient in ATP release also prevented the extravasation of RBCs in the lungs and their accumulation in the alveolar airspaces, suggesting that the ATP-sensitive adhesion of RBCs is an event upstream of their movement out of capillaries and into tissues. Interestingly, the strength of interactions between the RBC and ECs may be further fine-tuned following adhesion through regulation of subsequent ATP release (Leal Denis et al., 2013). Precisely how ATP prevents adhesion is uncertain, but could involve the stimulation of NO production by endothelial-NO synthase (eNOS) downstream of ATP binding to purinergic receptors on ECs (Erlinge and Burnstock, 2008).

## Mechanisms of ATP Export From Red Blood Cells

ATP export from RBCs can occur in response to various stimuli, and the underlying mechanisms and responsible signaling elements may vary (Keller et al., 2017; Kirby et al., 2021). Anucleate mature RBCs lack regulated pathways for the extrusion of cell-derived vesicles, and ATP export is therefore dependent on crossing cell membranes *via* transporters or possibly ion channels. Recently published lines of evidence suggest a role for pannexin 1 (Px1) as one component in the release of ATP from RBCs (Leal Denis et al., 2013; Kirby et al., 2021). We recently demonstrated the critical role of the ATP transporter Px1 in the ability of RBCs to export vasoregulatory ATP into the plasma *in vivo* and into the supernatant *in vitro* (Kirby et al., 2021). The Px1-dependent response appears to involve the (paradoxical) stimulatory action of the G protein Gi, in that the ATP-export response can be recapitulated by the Gi agonist mastoparan (Leal Denis et al., 2013; Kirby et al., 2021). The destination and activity of ATP exported from RBCs depend in part on the distribution and activity of ectonucleotidases (“ATPases,” for example CD73) present on cells and in soluble form in the circulation. Notably, we find that human RBCs have essentially no significant such activity. However, soluble CD73 is elevated in disease states, including sickle cell disease (Liu et al., 2018). As a consequence, the hydrolysis of extracellular ATP and accumulation of adenosine are favored, and signaling *via* adenosine receptors promotes deoxygenation of sickle hemoglobin and in turn (HbS) polymerization and RBC sickling. RBC ATP export is increased in hypoxia, and the resulting increases in plasma ATP in resistance vessels such as arterioles may contribute to hemodynamic responses to hypoxia. We recently demonstrated that in mice lacking the ATP exporter Px1, the hypotensive and hyperemic (blood flow-increasing) responses to hypoxia were significantly diminished, consistent with a role for ATP export in these responses (Kirby et al., 2021). We and others have reported that hypoxia increases the tendency of RBCs to adhere to ECs or basement membrane substrate proteins (Kirby et al., 2014). This increased adhesivity appears to be counterbalanced when RBC ATP is augmented, at least in the case of banked RBCs (Kirby et al., 2014; Kim et al., 2017). The specific vasoregulatory effects of ATP exported by Px1 may vary widely as a function of the cell type: although RBC-derived ATP appears to act as a vasodilator, Px1-mediated ATP export by smooth muscle cells can act in transduction of vasoconstrictor responses to alpha-adrenergic stimuli (Billaud et al., 2011).

## Coupling of Shear and Vasoactive Mediator Release

How does mechanical force, such as shear stress, lead to the production and release of vasoactive mediators? Mechanisms are still being unraveled, but new insight has resulted from investigation of the roles of mechanosensitive PIEZO1 channels. These cation channels enable intracellular Ca^++^ elevation. In ECs, Ca^++^ flux in response to either shear stress or a PIEZO1 agonist, Yoda, leads to cellular ATP release, followed by NO synthesis downstream of the actions of the exported ATP on purinergic receptors and endothelial NO synthase activation secondary to phosphorylation of eNOS by AKT (Wang et al., 2016). This signal transduction pathway appears to be important for the generation of flow-mediated vasodilation and blood pressure regulation.

## ATP Export From Red Blood Cells Couples Deformability and Modulation of Adhesivity

Recent scientific work has emphasized the power inherent in single-cell examination of function and molecular regulation, and this is exemplified in new insights into the behavior of RBCs in biology and medicine. Microfluidic handling of whole blood sampled from sickle cell disease patients revealed that the more deformable subset of SCD RBCs was, perhaps unexpectedly, the more likely to adhere (Alapan et al., 2014). This appears to be related to a tendency not to establish secondary foci of adhesion after an initial focal adhesion event takes place (Papageorgiou et al., 2018). On the other hand, the enhanced ability of SCD RBCs to adhere under conditions of hypoxia appears to involve the protrusion of HbS polymer fibers beyond the original boundary. This has the effect of increasing the cell surface area available for adhesion. Similarly, “knobby” (cellular-protrusion-bearing) erythrocytes infected with *Plasmodium falciparum* (an organism frequently responsible for malarial infection) displayed greater temperature-dependent binding to ECs than “knobless” erythrocytes infected with the same pathogen. Rather than a simple physical advantage in cell–cell binding, the proadhesive effect of the presence of knobs may reflect a localized abundance of adhesive ligands and receptors (Lubiana et al., 2020). Interestingly, Leal Denis and coworkers demonstrated that the export of ATP by RBCs made to adhere to coverslips coated with poly-D-lysine was far greater than that of RBCs on uncoated coverslips (Leal Denis et al., 2013).

## ATP and Red Blood Cell-Endothelial Adhesion in Thrombosis

Venous thromboses are composed in large part by RBCs, but the role of RBCs in clot formation (and resolution, if any) has been typically viewed as passive. RBC membrane disorders predispose to thrombosis (Andrews and Low, 1999), and following the export of ATP from RBCs, endothelial and leukocytic ectonucleotidases can hydrolyze the ATP to proaggregatory ADP (Hellem et al., 1961), activating platelets and driving clot formation. RBC adhesion may not always be pathogenic. Endothelial adhesion of RBC precursors may play an important and homeostatic role not only in venous thrombosis, but also in their ability to efficiently home in on supportive niches within the bone marrow microenvironment (Verfaillie, 1998). In addition to modulating the adhesion of RBCs to ECs, exported ATP may also act to limit endothelial permeability and thus inflammatory lung (and other organ) injury (Kolosova et al., 2008).

## Red Blood Cell Modulation of Neighbor Red Blood Cell Adhesivity

An important consequence of the recently recognized capacity of RBCs to export antiadhesive mediators is that the exported molecules can modulate not only the stickiness of the cell of origin, but also of neighboring cells. We showed, for example, that coincubation of SCD RBCs with fresh healthy (AA) RBCs attenuated the adhesivity of the SCD RBCs, whereas coincubation of AA RBCs stored for 30 days did not attenuate SS RBC adhesivity (McMahon et al., 2019).

## Pro-Inflammatory Actions of Extracellular ATP and Its Metabolites

In contrast to its homeostatic roles in vasoregulation, antiadhesive activity, and modulation of endothelial integrity *via* regulation of permeability (Kolosova et al., 2008), ATP can also act as a damage-associated molecular pattern (DAMP) through its action at the NLRP3 inflammasome on macrophages and other immune cells. Tumor necrosis factor-induced increases in endothelial permeability were dependent on Px1-mediated ATP export and CD39-mediated hydrolysis of ATP to adenosine (Maier-Begandt et al., 2021). In addition to hydrolysis to its precursors/metabolites, another means by which the levels of extracellular ATP are tightly regulated is through feedback inhibition of ongoing ATP export. This involves signaling of ADP at P2Y13 purinergic receptors on RBCs (Wang et al., 2005).

## Technical Note: Is It Exported ATP From Red Blood Cells, or Just an Artifact of Hemolysis?

The mechanisms and consequences of export of vasoactive mediators from RBCs can be modeled *in vitro*. Such models may use controlled gas exposure to mimic hypoxia, or controlled force to induce cell deformation. However, these reductionist experimental models are prone to artifact. In particular, bubbling of RBC suspensions can require substantial force and thereby promote the lysis of RBCs. The high sensitivity of mediator release from RBCs to mechanical force, such as deformation secondary to shear stress, has important implications for the conduct of investigation of the determinants of mediator release from RBCs in health and disease (McMahon, 2019). Specifically, the centrifugation commonly used to separate cellular from plasmatic components for assay can produce not only force-induced mediator export that dominates the extracellular space, but also lysis of vulnerable RBCs. When measuring extracellular ATP in such experiments, it is difficult or impossible to distinguish exported ATP from that which is generated by hemolysis. At a minimum, it is critical that the extent of RBC lysis be routinely reported in such experiments (Zhu et al., 2011; Sikora et al., 2014; Kirby et al., 2015, 2021). We also recommend that the observed extracellular mediator levels be compared to what would be expected with the observed lysis, taking into account the cellular and acellular volumes and the intracellular mediator levels. Unless extracellular mediator values are in clear excess over what *could be introduced* through the measured lysis, conclusions about export will not be possible or must be qualified. Complicating the problem is the fact that cell rupture can liberate not only vasoactive mediators like ATP, but also enzymes capable of eliminating these species (e.g., ATPases). We have discussed these important technical considerations in detail elsewhere, and recommended quality controls and a framework for interpretation and inter-laboratory comparisons. The centrifugation that is routinely used in order to separate RBCs from the other components of source whole blood, and to remove cell suspension buffers following application of pharmacological probes used to test mechanistic hypotheses, can itself deform the cells and elicit further, artifactual ATP release. In addition to eliciting deformation-induced release of mediators from RBCs, centrifugation can also promote the lysis of RBCs – particularly in the case of unhealthy RBCs – which also raises extracellular ATP levels and can be incorrectly ascribed to regulated ATP export.

Under conditions that favor its export from RBCs, plasma (or supernatant) ATP has been measured in the high-nanomolar to ∼1 μM range (Forrester and Lind, 1969; Ellsworth et al., 1995; Gonzalez-Alonso et al., 2002; Gorman et al., 2007; Wan et al., 2008; Zhu et al., 2011; Kirby et al., 2021). Notably, similar concentrations of infused ATP are well documented to elicit vasodilation and increases in regional blood flow, including in humans (Gonzalez-Alonso et al., 2002; Kirby et al., 2012). Typical levels of intraerythrocytic (ATP), by contrast, are in the range of hundreds of micromolar. Therefore, the lysis of greater than ∼0.2% of the RBC volume, whether in benchtop experiments or *in vivo*, could liberate enough ATP to mimic (or mask) that resulting from regulated, non-lytic release, depending on such additional variables as hematocrit, PO_2_, and the presence of ectonucleotidases (ATPases) (Kirby et al., 2015; McMahon, 2019). Interpretation of changes in extracellular ATP therefore need to take into account the extent of RBC lysis and the intra-RBC [ATP] among other factors.

## Other Vascular Mediators Exported by the Red Blood Cell: *S*-Nitrosothiols

ATP is not the only vasoregulatory mediator the RBC can export. RBC Hb is a well-studied site of S-nitrosylation, in which NO binds to a reactive cysteine (Cys) residue of a protein (in this case, Hb). This reaction is allosterically controlled and coupled, in that the transfer of a NO derivative from a heme group in Hb to the Cys is favored in the oxygenated, “R” conformation of Hb (Jia et al., 1996; Stamler et al., 1997). Conversely, the SNO moiety thus formed can be exported from the RBC upon deoxygenation, and this activity is favored *via* interaction between Hb and the membrane-resident protein AE1 (aka Band 3) (Sonveaux et al., 2007; McMahon, 2019). The AE1-Hb interaction is favored when Hb assumes the deoxy (“T”) conformation, and AE1-bound SNO might relay the exiting SNO (Pawloski et al., 2001). The final leg of the SNO export voyage appears to depend upon the action of the L-type amino acid transporter type 1 (LAT1), which is also recognized as playing a role in the import of small SNOs in some cell types (Li and Whorton, 2005, 2007). Once exported, SNO could act to cross-regulate the export of ATP by RBCs, since inhibitory S-nitrosylation of Px1 has been described. Conversely, exported ATP can act as an eNOS- and NO-dependent vasodilator (Olearczyk et al., 2004; Lohman et al., 2012; Ulker et al., 2018).

## Dysregulated Red Blood Cell ATP in Disease Settings

### ATP and Adenosine in Sickle Cell Disease

Sickle RBCs are characterized by elevated indices of oxidative stress and depressed ATP (Sabina et al., 2009), as well as elevated 2,3-DPG. See Table 1 for a summary of the changes in RBC ATP and related metabolites in disease settings and therapy, including examples of measured values. Zhang et al. (2011) identified elevated plasma adenosine as an important determinant of the increased DPG, which may contribute to SCD pathophysiology by decreasing O_2_ affinity, which in successive turn promotes HbS polymerization, RBC sickling, and hemolysis. The elevated plasma adenosine is believed to reflect both (a) increased plasma accumulation of ATP from RBCs themselves or tissues, and (b) activity of the ectonucleotidases CD39 and CD73, which hydrolyze ATP, leading ultimately to the formation of adenosine. Similar findings were demonstrated in mouse models of SCD and in blood from SCD patients. The effects of adenosine could be specifically inhibited by an antagonist of the A_2B_R adenosine receptor, suggesting a druggable process. Rogers et al. (2013) demonstrated abnormal binding of HbS to the RBC membrane in SCD, resulting in an impaired ability of SCD RBCs to sequester the glycolytic EMP enzymes. Consequently, HMP activity is suppressed, leading to decreased ability to generate NADPH and maintain glutathione in the reduced state. Together, these deficits permit the accrual of oxidative damage to proteins and lipids, both basally and in response to an oxidative challenge.

**TABLE 1:** Changes in RBC function and RBC ATP in human disease.

**Disease or Condition**	**RBC functional change**	**Change in RBC ATP, and in other RBC organic phosphates**	**RBC or plasma [ATP]**	**Mechanism of RBC ATP change (proposed)**	**Reversibility of the RBC ATP change**	**References**
Sickle cell disease	Susceptible to sickling; poorly deformable; increased adhesivity; decreased vasoactivity	Decreased; increased RBC DPG	RBC ATP: ∼6.2 vs. 5.4 μmol/g Hb (Sabina et al., 2009)	Oxidative damage to metabolon and AE1	Reversible (from 320 to 380 μg/mL after PKR activator) (Kalfa et al., 2019)	Sabina et al., 2009; Rogers et al., 2013; Kalfa et al., 2019
G6PD deficiency	Susceptible to RBC lysis	None: preserved intra-RBC ATP; decreased NADPH	Not stated (arbitrary units only)	Antioxidant depletion	n/a	Francis et al., 2020
Sepsis	Poorly deformable; increased adhesivity	Decreased	RBC ATP: 60.7 μM septic neonates vs. 71.6 μM non-septic neonates in ICU	Multifactorial	Undetermined	Li et al., 2019
COVID-19	Undetermined	Trend toward increase (*p* = 0.07)	Not stated (arbitrary units only)	Unknown	Undetermined	Thomas et al., 2020a
RBC storage lesion	Poorly deformable; increased adhesivity; decreased vasoactivity; decreased survival	Late decline in intra-RBC ATP; progressive decline in ATP export capacity	RBC ATP: from 0.26 basally to 0.20 mol ATP/mol Hb at 42 days’ storage (Hogman et al., 2006); RBC ATP export: from 250 nM at Day 0 to 52 nM on Day 28	Unknown; oxidative lesion?	The fall in RBC ATP is reversible with PIPA (1.7–3.6 μmol/g Hb) (Kirby et al., 2014) or preventable via near-anaerobic storage (∼2.2–3.2 μmol/g Hb) (Yoshida et al., 2017)	Hogman et al., 2006; Zhu et al., 2011; Kirby et al., 2014
Diabetes mellitus	Increased adhesivity; decreased vasoactivity	Decreased RBC ATP export	RBC ATP export: ∼53 vs. 220 nM from healthy RBCs (Campanella et al., 2005)	Protein glyc(osyl)ation; reduced G_i_ abundance	Undetermined	James et al., 2004; Sprague et al., 2006; Subasinghe and Spence, 2008
Pyruvate kinase deficiency (PKD)	Hemolytic anemia	Decreased RBC ATP; increased 2,3-DPG		PKD	Reversible: RBC ATP rose 1.6-fold after PK activation (mean) (Chu et al., 2008)	Rab et al., 2021; Shrestha et al., 2021

*Putative mechanisms, examples of published measurements, and reversibility (response to therapy) are also indicated.*

### Red Blood Cells From Volunteers With Glucose-6-Phosphate Dehydrogenase Deficiency

Red blood cells from volunteers with glucose-6-phosphate dehydrogenase deficiency were significantly inferior to RBCs from G6PD-competent donors with respect to post-transfusion recovery (a measure of RBC survival in the circulation), although the difference was small. This functional RBC storage lesion correlated with significant defects in glutathione (a central molecular player in cellular redox capacity), increased purine oxidation, and increased glycolysis (an expected shift in G6PD deficiency). When the G6PD inhibitor DHEA was used to decrease NADPH formation in RBCs, ATP export in response to deformation was also suppressed (Subasinghe and Spence, 2008). Taken together, these findings underscore the importance of the dynamic balance between glycolysis (EMP, generating critical metabolites such as ATP) and the HMP pathway (generating the critical reducing equivalent, NADPH necessary for full glycolytic flux) in the RBC. These data also point to the potential for leveraging knowledge of existing genetic disorders to improve the understanding of function-metabolite relationships in RBC transfusion medicine.

### Anemia in COVID-19

Moderate or severe COVID-19 disease is a form of sepsis, even though it is infrequently labeled as such, and COVID-19 may share pathophysiologic elements and sequelae with other sepsis etiologies. Pre-existing anemia is a risk factor for COVID-19 infection and severity according to some studies. In a report on comorbidities among COVID-19 patients in Wuhan, the original epicenter of what became the COVID-19 pandemic, the odds ratio for severe illness was 3.47 (95% CI: 1.02–11.75, *n* = 222) for patients with anemia (Tao et al., 2020). Guan et al. (2020) reported that although frank anemia was not prevalent in their cohort (*n* = 1099), Hb levels were significantly lower in patients with severe illness than in those with non-severe COVID-19 disease. Modest but progressive declines in Hb values were statistically significant in ill COVID-19 patients, and morphologically abnormal RBCs such as stomatocytes and knizocytes were observed in the blood of COVID-19 patients (Berzuini et al., 2021). Given the crucial role of RBCs in O_2_ transport and in thrombosis, the role of RBC mediator dysregulation in COVID-19 complications requires investigation, including study of interaction with the known dysregulation of banked RBCs. The apparent association between (ABO) blood group antigens in COVID-19 and clinical outcomes suggests that the possibility of increased RBC adhesivity in this disease deserves further investigation (Leaf et al., 2020; Zietz and Tatonetti, 2020; Zhao et al., 2021). Potential RBC transfusate or systemic remedies also deserve study in preclinical COVID-19 models.

The COVID-19 pandemic profoundly influenced the study of “viral sepsis,” as intensive care units (ICUs) saw record numbers of cases over the last two years. The subsequent rush to biobank samples for molecular phenotyping has been unprecedented. Plasma and serum, which are easy to obtain, were the focus of dozens, if not hundreds, of ‘omic (e.g., metabolomics and proteomics) studies of COVID-19 disease, including mass spectrometry-based proteomics and metabolomics (Chen et al., 2020; D’Alessandro et al., 2020; Hou et al., 2020; Messner et al., 2020; Shen et al., 2020; Thomas et al., 2020b; Zecha et al., 2020; Aggarwal et al., 2021; Overmyer et al., 2021; Sindelar et al., 2021; Sullivan et al., 2021). Changes in metabolite and protein expression are now well-correlated with COVID-19 severity, and this approach may facilitate the identification of diagnostic and prognostic markers of disease, yield mechanistic insight and reveal potential therapeutic targets. However, whole blood or RBCs have been largely ignored in COVID-19 for proteomics or metabolomic profiling. A single study applied ‘omic approaches to isolated RBCs in COVID-19 (Thomas et al., 2020a), showing alterations in numerous metabolite classes, including free fatty acids, acylcarnitines and sphingolipids.

### Metabolic and Other Changes in COVID-19 Red Blood Cells

COVID-19 RBCs displayed increased glycolytic intermediates, signatures of oxidation and fragmentation of key structural and functional proteins including ankyrin, spectrin beta, and band 3 (AE1), as reported in a multi-omic investigation (Thomas et al., 2020a). These metabolomic and proteomic findings can be mutually rationalized in part by the observation that the evident damage to band 3 (AE1) is expected to untether the glycolytic enzyme complex or “metabolon” from its function-suppressing assembly at band 3. With glycolysis favored, the hexose monophosphate (HMP) pathway responsible for generating the reducing equivalents NADPH and, in turn, glutathione, is compromised in part *via* limitation of the substrate glucose-6-phosphate. Systemic changes in metabolic control are also present in patients with COVID-19, and may persist well beyond the acute illness. Not only is diabetes an important comorbidity predisposing patients to COVID-19 infection, but insulin resistance was also detected in a substantial fraction of non-diabetic COVID-19 patients, even among normoglycemic patients (Montefusco et al., 2021). Moreover, hyperglycemia was present in over of 30% of “recovered” patients at least 2 months later. The mechanisms underlying the systemic alterations in glycometabolic control, and those in RBC metabolites specifically, remain undetermined. The functional significance of the metabolic and other changes in COVID RBCs is under investigation, but depressed RBC deformability, even compared to other sepsis patients, has been reported (Renoux et al., 2021).

### Red Blood Cell ATP as an Index of Red Blood Cell Stress and Fate

In nucleated cells, it is now clear that the regulation of metabolic checkpoints by growth factors in turn governs cellular fate direction, such as programmed cell death (apoptosis) or proliferation or dedifferentiation. In the anucleate RBC, there is still a substantial role for metabolic shifts in linking cell stress with either survival or with safe and accelerated clearance from the circulation. Specifically, RBCs rendered dysfunctional through nutrient, temperature, or osmotic stress can nevertheless avoid lysis (which *via* the liberation of free heme, hemoglobin, and iron can be highly toxic) (Nemkov et al., 2020). Such cells can instead be tagged for splenic (extravascular) elimination by externalization of phosphatidyl serine (PS) in the cell membrane. PS exposure marks the RBC for clearance, and is the net result of the action of ATP-dependent flippases that act basally to keep PS oriented mainly inward and scramblases that promote its outward orientation of PS. Cell stress tending to deplete the intraerythrocytic ATP necessary to operate the flippase may also compromise the ability of RBCs to exclude calcium. As RBCs then admit Ca^++^, scramblase activity ensues. Increases in plasma ATP in older humans are depressed vs. those of healthy younger subjects (Kirby et al., 2012). While the mechanism is unknown, isolated RBCs from these same subjects display a similar age-dependent depression of the ability to export ATP.

### Red Blood Cell Transfusion and ATP

The widely reproduced finding that restrictive RBC transfusion is non-inferior to liberal RBC transfusion in patients with anemia, taken together with the finding that anemia itself is a strong risk factor for poor outcomes, suggests that there is potential for improved approaches to RBC storage in anticipation of transfusion (Corwin et al., 2004). Evidence from individual studies and from a meta-analysis indicates that RBCs banked for later transfusion raise not only bulk O_2_ delivery but also O_2_ consumption by the tissues (Cavalcante Dos Santos et al., 2020). But this bulk increase in O_2_ delivery does not necessarily result in a net improvement in organ function in vulnerable patients, possibly reflecting storage-induced loss of the regulation of O_2_ delivery *distribution*. RBC banking may compromise not only O_2_ delivery distribution, but also O_2_ uptake by RBCs in the lung. RBCs transfused after storage paradoxically and significantly depress O_2_ uptake in critically ill adult patients. Turgeman et al. (2020) showed that blood O_2_ saturation and arterial PO_2_ measured 24 h after RBC transfusion fell relative to baseline. We demonstrated similar O_2_ uptake deficits in nude mice receiving transfusion of stored human RBCs (Zhu et al., 2011; Riccio et al., 2015).

Currently used RBC additive solutions were designed with ATP preservation in mind. But our finding that the ability of stored RBCs to export vasoregulatory ATP declines progressively with storage time (Zhu et al., 2011) suggests the possibility that clinical outcomes after RBC transfusion could be improved *via* optimization of the blood flow component of the O_2_ delivery formula. Indeed, the United States Food and Drug Administration and the (United States) National Institutes of Health have recognized the need for updated approaches to the licensing of RBC products that incentivize the goal of improved clinical benefits in anemic patients receiving transfusion (Vostal et al., 2018). There is also recognition by both regulatory authorities and the medical and scientific communities of the need for improved approaches for assessing tissue oxygenation as a function of RBC transfusion (Ochocinska et al., 2021).

### Red Blood Cell Storage Duration and Trauma

Retrospective studies had indicated an increased risk of poorer outcomes following transfusion with longer-stored RBCs in trauma, ICU, and cardiac surgery patients. Subsequent randomized controlled data demonstrated that RBCs stored longer periods (roughly 3–5 weeks) were non-inferior to fresher RBCs (e.g., less than 7 days) in ICU and cardiac surgery patients among others (Lacroix et al., 2015; Steiner et al., 2015), but this has not been carefully studied in trauma. The significant RBC changes during blood banking may compound those seen in trauma patients, and include morphological changes, alterations in adhesivity, and other maladaptive or injurious cell–cell interactions. These changes can compromise blood (RBC) flow and shorten RBC survival. Similar lesions are observed in RBCs stored for transfusion, and therefore transfused RBCs might exacerbate the existing micro- and macro-hemodynamic changes in trauma patients. Among 15 clinical studies of RBC transfusion in trauma (summarized in Sparrow, 2015), 93% reported adverse events with transfusion of longer-stored RBCs. These longer stored units were associated with increased risk of infection, organ failure, vascular complications and, in some studies, higher mortality. There are currently no data from randomized, controlled clinical trials (RCTs) available regarding the effect of RBC storage duration in trauma patients with hemorrhage. However, given that after longer storage times RBCs are more prone to being phagocytosed, are more fragile, may promote vascular permeability, demonstrate increased NO scavenging, and display increased adhesivity to ECs (Zhu et al., 2011), there is reason for concern over unfavorable physiologic changes following longer-stored RBC transfusion. Still, it has not yet been determined whether these *in vitro* findings actually translate into inferior clinical outcomes. Further research, particularly RCTs in human patients, will be needed in order to draw more substantial conclusions regarding RBC storage duration in the setting of trauma. Meanwhile, investigation of improved approaches to RBC-based resuscitation during hemorrhagic shock is taking place in animal models. In a rat hemorrhagic model of acute anemia, transfusion of anaerobically stored RBCs was superior to that of conventional RBCs in that a lower RBC volume was required in order to normalize hemodynamics (mean arterial blood pressure), a study endpoint chosen because of its adoption in clinical guidelines for trauma management including those for Advanced Trauma Life Support and from the American College of Surgeons (Williams et al., 2020). The metabolic and functional profile of RBCs stored under anaerobic conditions is discussed below.

### Diabetes and Red Blood Cell ATP Export

James and coworkers demonstrated impairment in the ability of diabetic human RBCs to vasodilate blood vessels. The impairment was positively associated with the extent of Hb glycosylation, and inversely associated with levels of vasodilator SNOs (James et al., 2004). Subasinghe and Spence (2008) demonstrated that the ability of human diabetic RBCs to export ATP is depressed. Given the close interplay between ATP generation and G6PD-regulated redox status in the RBC, this is perhaps not surprising. Sprague and coworkers also demonstrated depressed ATP export from diabetic human RBCs, in parallel with reduced abundance in the RBCs of the G-protein (G_i_) implicated in transducing regulated ATP export (Sprague et al., 2006).

## Therapeutic Approaches to Modify Red Blood Cell ATP Content And/Or Export

### Augmenting ATP and 2,3-DPG in Red Blood Cells Used for Exchange Transfusion in SCD Patients

**FIGURE 2:**
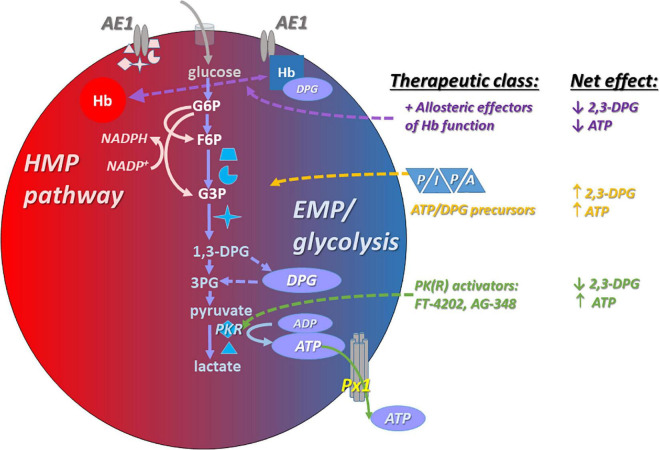
Interventions that increase ATP content in RBCs typically also promote the capacity for increased ATP export basally and in response to stimuli such as hypoxia. Among these interventions are post-RBC-storage (pre-transfusion) *exposure to PIPA* (yellow), a commercially available and FDA-approved solution containing phosphate, inosine, pyruvate, and adenine (aka Rejuvesol). PIPA use effectively loads the RBC with the substrate necessary to synthesize *ATP*). Other *ATP*-augmenting approaches include anaerobic storage (preserving *ATP;* not shown), and *PK(R) activators* (green), which may be administered orally/systemically or at the RBC transfusate (blood product unit) level. PKR activation raises *ATP* levels at the expense of *2,3-DPG* levels. Finally, *allosteric modulators (purple)* can influence RBC ATP content by influencing the propensity of Hb (hemoglobin) to bind to AE1 (displacing the EMP metabolon), and can influence the ability of RBCs to export *ATP via* modulation of oxygenation status.

Gehrke et al. (2019) investigated the influence of RBC unit exposure to pyruvate, inosine, phosphate, and adenine (PIPA) solution on metabolite profiles in the blood of sickle cell disease patients (a RBC transfusate treatment schematized in Figure 2). Increases of the antioxidant molecules glutathione and NADPH and the NO precursors arginine and citrulline were seen, as well as the expected increases in RBC ATP and 2,3-DPG (Gehrke et al., 2019). Alternatively, it may be desirable in SCD to raise ATP levels (needed for basal and inducible ATP export, and for numerous intracellular homeostatic actions) without elevating DPG levels, or even while reducing DPG content. The rationale is that elevated DPG in SCD is maladaptive in that it lowers O_2_ affinity in the SCD RBC. This in turn favors HbS deoxygenation, which in turn promotes the Hb polymerization that results in RBC sickling.

### Pyruvate Kinase Activation and Red Blood Cell ATP

Patients with pyruvate kinase deficiency (PKD) are susceptible to hemolytic anemia (Table 1) because RBC PK is critical for ATP generation, given the absence of mitochondria in mature RBCs. Deficient ATP in the RBCs of patients with PKD (Figure 2) can be rescued with a non-specific pyruvate kinase activator AG-348, also known as mitapivat (Rab et al., 2021). These effects are accompanied by increases in the enzyme’s temperature-dependent stability and activity. Responses were determined in part by the baseline level of PK, with poor responses among PKD patients having low PK abundance. Functionally, the augmented ATP was associated with improved deformability of the RBCs, and AG-348 also increased ATP and PK activity in the RBCs of healthy control subjects. RBC ATP production can be elevated in healthy or SCD RBCs by activation of an RBC-specific pyruvate kinase (PKR), and in this case the augmented ATP production (*via* glycolysis) takes place at the expense of DPG. Shrestha et al. (2021) demonstrated that SCD-modeling “Berk” mice given the orally available PKR activator FT-4202 (etavopivat) for 2 weeks had significantly increased ATP and reduced 2,3-DPG, improved deformability, lower susceptibility to deoxygenation-induced sickling and rigidity, and attenuated indices of hemolysis.

### Metabolic and Functional Consequences of Anaerobic Red Blood Cell Storage

One promising alternative approach to improve outcomes following the transfusion of stored RBCs focuses on the avoidance of accumulation of oxidative lesions in the banked RBC. Depletion of antioxidant substrates and decline of enzymatic activities occur during storage of RBCs, and the conventionally stored RBC unit encounters progressively more abundant oxygen – and attendant oxidation – because the gas-permeable RBC unit storage bag allows the gradual entry of oxygen. Interestingly, there was marked variability in the initial oxygenation state (defined as the percentage saturation of Hb with O_2_) in leukoreduced RBC units obtained from each of several independent blood product suppliers and then stored in AS-3 additive solution (Yoshida et al., 2017). Given the centrality of O_2_-driven oxidative chemistry in the progressive deterioration of RBC function during RBC storage, it is reasonable to hypothesize that the variability in RBC oxygenation contributes to variation in RBC unit quality. Yoshida et al. (2017, 2019) demonstrated protection of RBCs from the set of oxidative lesions with the use of anaerobic storage of otherwise conventionally processed and stored units. Specifically, anaerobic storage protected against declines in ATP and 2,3-DPG levels, slowed rates of hemolysis, and prevented oxidation of lipids and hemoglobin while preserving the antioxidant glutathione (GSH). The degree of the protection from these lesions correlated with the magnitude of reduction in SO_2_. The authors also showed improved RBC deformability using this storage method. Some of these beneficial effects of anaerobic storage of RBCs appear to depend not only on minimizing O_2_ and thus reactive oxygen species (ROS), but also on the parallel CO_2_ elimination effected by the sparging (gas flushing) used to deoxygenate the RBCs. When RBCs were stored under isocapnic conditions under which CO_2_ concentration (and secondarily, pH) was held steady, DPG declined at rates similar to those in conventionally stored RBCs (Dumont et al., 2016).

### Allosteric Modulation of Hemoglobin Function and Its Interaction With Vasoactive Mediators

Voxelotor, a positive allosteric modulator, represents an important proof-of-principle that targeting the position and poise of the Hb oxygen equilibrium curve (OEC) in sickle cell disease can safely convey a net clinical benefit (Vichinsky et al., 2019). “Positive” allosteric effectors that promote the O_2_ affinity of Hb will tend to disfavor glycolysis and thus 2,3-DPG and ATP production (as schematized in Figure 2). More recently, Abdulmalik et al. (2020) have advanced this therapeutic category by developing and testing multi-functionality agents that not only act as classical allosteric modulators (whose action is oxygenation state-dependent), but also act downstream to directly prevent the polymerization of HbS. A third action can also be achieved through functionalization of the parent aldehyde with a nitroso side group (Xu et al., 2015). The latter functionality could be leveraged to promote O_2_ delivery *via* RBC export of SNOs even while O_2_ offloading from Hb itself is braked.

## Conclusion

The regulated production of key RBC metabolites, including vasoregulatory ATP, the allosteric effector DPG, and the reductant NADPH, is sensitive to cellular cues unique to the RBC, namely its high oxidant load (*via* reactive oxygen species) and its mission of efficient O_2_ delivery and CO_2_ clearance. Glycolysis generates ATP and DPG preferentially when RBCs have offloaded O_2_, coinciding with the O_2_ delivery limb of the RBC circuit, *via* both bulk O_2_ transfer and blood flow control. Disordered generation of these metabolites characterizes several health conditions and disease states. New and repurposed therapeutic approaches can drive the generation and export of ATP and are worthy of investigation.

## Dedication

This paper is dedicated to the memory of our friend and collaborator, Joseph Bonaventura, Ph.D. (1942–2021).

## Author Contributions

TM conceived the manuscript. All authors participated in researching, writing, and editing the manuscript.

## Conflict of Interest

TM received research funding from Hemanext LLC. A grant with Forma Therapeutics to TM is pending. The remaining authors declare that the research was conducted in the absence of any commercial or financial relationships that could be construed as a potential conflict of interest.

## Publisher’s Note

All claims expressed in this article are solely those of the authors and do not necessarily represent those of their affiliated organizations, or those of the publisher, the editors and the reviewers. Any product that may be evaluated in this article, or claim that may be made by its manufacturer, is not guaranteed or endorsed by the publisher.
